# *Chlamydomonas angulosa* (Green Alga) and *Nostoc commune* (Blue-Green Alga) Microalgae-Cellulose Composite Aerogel Beads: Manufacture, Physicochemical Characterization, and Cd (II) Adsorption

**DOI:** 10.3390/ma11040562

**Published:** 2018-04-05

**Authors:** Kyojung Hwang, Gu-Joong Kwon, Jiwook Yang, Minyoung Kim, Won Joung Hwang, Wonjae Youe, Dae-Young Kim

**Affiliations:** 1 Department of Biological and Environmental Science, Dongguk University-Ilsan, Biomedical Campus, Goyang-si, Ilsandong-gu 10326, Korea; kyojung7@naver.com (K.H.); gjkwon@dongguk.edu (G.-J.K.); jwjii0@naver.com (J.Y.); minmiy@hotmail.com (M.K.); 2 Division of Wood Processing, Department of Forest Products, National Institute of Forest Science, 57 Hoegiro, Dongdaemun-gu, Seoul 02455, Korea; hwjgreen@naver.com; 3 Division of Wood Chemistry & Microbiology, Department of Forest Products, National Institute of Forest Science, 57 Hoegiro, Dongdaemun-gu, Seoul 02455, Korea; ulrin@naver.com

**Keywords:** cellulose, microalgae, composite aerogel beads, Cd (II), adsorption

## Abstract

This study presents composite aerogel beads prepared by mixing dissolved cellulose with *Chlamydomonas angulosa* and *Nostoc commune* cells, respectively, at 0.1, 0.3, and 0.5% (*w*/*w*). The manufactured composites (termed regenerated cellulose (RC)), with *C*. *angulosa* (RCCA-(1, 3, and 5)), and with *N*. *commune* (RCNC-(1, 3, and 5)) were analyzed. Both RCCA-5 and RCNC-5 showed the high specific surface area to be about 261.3 and 332.8 m^2^·g^−1^. In the microstructure analysis, network structures were observed in the cross-sections of RC, RCCA-5, and RCNC-5. The pyrolysis temperature of the RCCA-5 and RCNC-5 composite aerogel beads was rapidly increased about 250 °C during the mixing of cellulose with *C*. *angulosa* and *N*. *commune*. The chemical analysis of RC, RCCA-5, and RCNC-5 showed peaks corresponding to various functional groups, such as amide, carboxyl, and hydroxyl groups from protein, lipid, and carbohydrate. RCNC-5 at pH 6 demonstrated highest Cd^2+^ removal rate about 90.3%, 82.1%, and 63.1% at 10, 25, and 50 ppm Cd^2+^, respectively. At pH 6, Cd^2+^ adsorption rates per unit weight of the RCNC-5 were about 0.9025, 2.0514, and 3.1547 mg/g at 10, 25, and 50 ppm, respectively. The peaks assigned to the amide, carboxyl, and hydroxyl groups in RCCA-5, RCNC-5, and RC were shifted or disappeared immediately after adsorption of Cd^2+^. The specific surface area, total pore volume, and mean pore diameter of composites was decreased due to adsorption of Cd^2+^ on the developed materials. As can be seen in the X-ray powder diffraction (XRD) spectrum, significant changes in the molecular structure of the composite aerogel beads were not observed even after adsorption of Cd^2+^.

## 1. Introduction

Heavy metals are widely used in various sectors, due to their role in various processes and modern industrial applications [1]. As the demand increases, heavy metal pollution of water becomes increasingly problematic, due to their toxicity, non-biodegradability, and persistence in the environment [2]. Although trace amounts of copper, zinc, and iron are essential for the metabolism of life, mercury, cadmium, and lead are crucially harmful [3]. Moreover, excess amounts of the essential minerals and prolonged exposure to heavy metals has been associated with various diseases, such as neurotoxicity and lung cancer.

Cadmium is used in numerous industries for electroplating, manufacture of nickel–cadmium batteries, and in paint pigments and polyvinyl chloride plastics [4,5,6]. For the disposal of heavy metals, like cadmium, biological treatments [7,8], physicochemical treatments [9,10], ion-exchange [11], membrane filtration [12], and adsorption is used [13,14,15]. However, physical treatments are time-consuming and costly [16], while chemical treatments require numerous, expensive chemical substances and cause secondary pollution [17]. Alternatively, these issues can be avoided by using agricultural products and by-products as absorbents. For instance, coconut shell, orange peel, rice husk, peanut husk, and sawdust are effective in reducing the concentration of heavy metals [18,19,20,21]. Nonetheless, this method requires a second procedure to efficiently separate the metal-loaded biomass. In this context, new technologies are being studied.

Cellulose is the most abundant and sustainable organic polymer resource available worldwide. Regenerated cellulose fibers possess many exposed hydroxyl groups, giving the molecule its characteristic properties of hydrophilicity, degradability, and chemical reactivity. In addition, the pore inside aerogel does not change from the liquid to air substitution, giving the aerogel structural stability and high specific surface area [22]. Many solvents like NaOH/Cs_2_, lithium chloride/N, N- dimethyl acetamide (LiCl/DMAc), N-methylmorpholine-N-oxide (NMMO), LiOH/urea, and NaOH/urea were developed for dissolution of cellulose. Among these, LiOH/urea and NaOH/urea are very economical due to the solvent’s cheap price [23]. Notably, the dissolution and regeneration of cellulose in aqueous NaOH/urea or LiOH/urea solutions constitute an effective strategy to generate cellulose aerogels with potential use as absorbents [24], considering the abundance of hydroxyl groups exposed in the regenerated polymer [25]. Nonetheless, the hydroxyl groups of cellulose have limited absorbing capacity [26]. Consequently, there are many studies on carboxymethyl cellulose (CMC), which is functionalized by chemical modification with carboxymethyl to increase its adsorption capacity. In addition, investigations on designing and developing composites from bio-waste resources are undergoing, but further research is needed.

Microalgae include not only eukaryotic algae but also cyanobacteria. Microalgae fix carbon dioxide (a greenhouse gas) and can produce biomass gas and various multifunctional bioactive compounds. They can also synthesize useful substances, such as vitamins and pigment [27,28]. *Chlamydomonas angulosa*, a green microalgal species, is a useful biological resource and a promising tool for heavy metal bioremediation [29]. *Nostoc commune* is a widely distributed, atmospheric N_2_-fixing cyanobacteria. It has traditionally been used in China as food and medicine [30,31]. Microalgae that display these advantages are being intensively studied as sources of renewable energy, pigments, and production systems of bioactive compounds. However, their use as a composite is inadequate.

Therefore, the current study investigated the use of cellulose and microalgae to reduce the concentration of heavy metals in water effectively. Cellulose was dissolved in LiOH/urea/H_2_O solution. Then, *C*. *angulosa* (green alga) and *N*. *commune* (blue-green alga or cyanobacteria) were synthesized as composite aerogels, by combining various proportions of dissolved cellulose with *C*. *angulosa* and *N*. *commune*, respectively. The physical traits of the aerogels were compared by specific surface area. Scanning electron microscopy (SEM), thermogravimetric analysis (TGA) and Fourier transform infrared (FTIR) spectroscopy were conducted to evaluate the chemical traits. Additionally, the composite aerogels were mixed with cadmium to establish their adsorption capacity, according to the heavy metal concentration, pH and time. The absorbed cadmium was calculated, using inductively coupled plasma spectroscopy (ICP). Based on the analysis, the potential of the composite aerogels as an absorbent was discussed. After cadmium adsorption of the composite aerogel beads, a characteristic analysis was carried out using specific surface area and X-ray diffractometer (XRD) to confirm the variation in the composite aerogel beads.

## 2. Materials and Methods

### 2.1. Materials

Cellulose was derived from the Whatman filter paper (grade 5; Cat. No. 1005110, Whatman International, Berkhamsted, UK). Lithium hydroxide monohydrate (LiOH), urea (CH_4_N_2_O), and methyl alcohol (CH_3_OH) were used for dissolution and regeneration of cellulose from the filter paper. The internal pores of regenerated cellulose were substituted with ethyl alcohol (C_2_H_5_OH) and *tert*-butanol (C_4_H_10_O). Diluted cadmium standard solution (Kanto Chemical Co., Inc., Tokyo, Japan) was used for the adsorption test. Microalgae cultures were supplied by the Korea Marine Microalgae Culture Center (KMMCC) in City, South Korea. Jaworski’s medium (JM) (Composition (g/L): Ca(NO_3_)_2_·4H_2_O (20.00), KH_2_PO_4_ (12.40), MgSO_4_·7H_2_O (50.00), NaHCO_3_ (15.90), EDTA-FeNa (2.25), EDTA-Na_2_ (2.25), H_3_BO_3_ (2.48), MnCl_2_·4H_2_O (1.39), (NH_4_)_6_Mo_7_O_24_·4H_2_O (1.00), cyanocobalamin (0.04), thiamine HCl (0.04), biotin (0.04), NaNO_3_ (80.00), and Na_2_HPO_4_·12H_2_O (36.00)) were used as a culture medium.

### 2.2. C. angulosa and N. commune Growth Conditions

*C*. *angulosa* (green alga) and *N*. *commune* (blue-green alga) were placed in an Erlenmeyer flask with stock solution and distilled water, to obtain a final volume of 2 L. The system was operated as a continuous culture, with aeration supplied by a compressor (Air-4000, Resun, Shenzhen, China) at 0.1 L·min^−1^. Illumination was provided at a photosynthetic photon flux density (PPFD) of 39 µmol m^−2^·s^−1^ under 16 h light/8 h darkness cycle, for 4 weeks at 22 °C. Cultured microalgae were washed with distilled water and centrifuged (3500 rpm, 25 min). The powder was obtained from the centrifuged material by freeze-drying.

### 2.3. Preparation of Cellulose Solution

The filter paper was cut into 10 × 10 mm^2^ pieces and ground to a powder (7010G Blender, Waring Commercial, McConnellsburg, PA, USA) at high speed (22,000 rpm) for 5 min (×2). LiOH/urea/H_2_O solvent at 8:12:80 (*w*/*w*/*w*) was prepared at –13 °C, in a refrigerated temperature bath (TC-500, Brookfield Engineering Laboratories, Inc., Middleboro, MA, USA). The cellulose powder was dissolved in the chilled solvent at 2% (*w*/*w*) for 25 min [32].

### 2.4. Microalgae-Cellulose Composite Aerogel Beads Manufacture

The microalgae-cellulose composite ratios are shown in Table 1. LiOH/urea/H_2_O-dissolved cellulose was mixed with *C*. *angulosa* ((0.1, 0.3, and 0.5% (*w*/*w*)) and *N*. *commune* (0.1%, 0.3%, and 0.5% (*w*/*w*)), respectively, at 300 rpm under room temperature. Gelation was induced by the drop wise addition of methyl alcohol. The gel was regenerated for 12 h. Regenerated composite gel beads were washed with running water to eliminate LiOH and urea from the internal structure. The pores inside the gel beads were substituted with ethyl alcohol (1 h, three times) and *tert*-butanol (1 h, three times), and then freeze-dried for 72 h. The organic solvent was used to maintain disposed structure at the drying process [33].

### 2.5. Characterization of Manufactured Microalgae-Cellulose Composite Aerogel Beads

#### 2.5.1. Brunauer–Emmett–Teller (BET) Surface Area Measurement

The microalgae-cellulose composites were dried at 80 °C for 3 h. Then, the structural characteristics of the composites were determined by nitrogen gas adsorption at 77 K, using a BET surface area and pore size analyzer (Belsorp-mini II, BEL Japan Inc., Osaka, Japan). Specific surface area (*S*_BET_), total pore volume (*Vt*), and pore diameter (*Dp*) of the composites were calculated by the nitrogen adsorption isotherm. Total pore volume was computed from the data at a relative pressure (*P*/*P_O_*) of 0.99. Average pore volume was calculated using the following formula [34,35]:

*Dp* (nm) = 4*Vt* × 1000/*S*_BET_. (1)

The composites with superior BET and pore characteristics were selected for further analysis (Section 2.5.2, Section 2.5.3, Section 2.5.4, Section 2.5.5, Section 2.5.6 and Section 2.5.7).

#### 2.5.2. Structural Analysis

Morphological characteristics of the composites were observed using SEM (EM-30, Coxem, Daejeon, Korea) at 20 kV acceleration voltage. The composites were cut into cross-sections using a microtome blade (MB35 Premier, Thermo Scientific, Waltham, MA, USA). The surface and cross-section of the composites were sputter-coated (KIC-1A, Coxem, Korea) with Au at 5 mA, before visualization.

#### 2.5.3. Thermoanalysis

A Q600-SDT thermoanalyzer (TA Instruments Inc., Newcastle, DE, USA) was used to evaluate the thermal properties of the raw materials and composites, which were pre-dried at 105 °C for 24 h to remove moisture. Afterwards, the samples were heated from 0 to 600 °C min^−1^. The weight decrease was measured as a function of increasing temperature.

#### 2.5.4. Chemical Characterization

The chemical properties of the raw materials and composites were assessed using attenuated total reflection ATR-FTIR spectroscopy (Vertex 40, Bruker Optics, Bremen, Germany) over the wavelength range of 600 to 4500 cm^−1^.

#### 2.5.5. Cd^2+^ Adsorption Test

Cd^2+^ adsorption by the manufactured composites, RC, RCCA-5, and RCNC-5, was tested as a function of Cd^2+^ concentration (10, 25, and 50 ppm), adsorption time (5, 10, 30, 60, and 120 min), and pH (4, 5, and 6). Ten milliliters of Cd^2+^ aqueous solutions (at the various concentrations and pH) were reacted with 0.1 g of RC, RCCA-5, and RCNC-5, respectively, at 25 °C, under constant agitation at 150 rpm in a shaking incubator (Wis-20R, Wisecube Co., Ltd., Seoul, Korea). Next, the reaction solution was filtered through a disposable membrane filter (pore size 0.45 µm, Advantec, Tokyo, Japan). The concentration of residual Cd^2+^ in the solution was determined using ICP (Prodigy ICP, Teledyne Leeman Labs Co., Hudson, NH, USA), and the data was evaluated as follows:

(2) $$\mathrm{Removal}\;\mathrm{rate}:\;q\;\left(\%\right)=\frac{\left(Co-C\right)\times 100}{Co},$$

(3) $$\mathrm{Adsorbing}\;\mathrm{capacity}:\;Q=\frac{\left(Co-C\right)\times V}{m},$$

where *q* is the Cd^2+^ removal rate, *Q* is the Cd^2+^ adsorbing capacity by the composite aerogel beads (dry weight: mg·g^−1^), *Co* is the concentration of Cd^2+^ in the stock solution (mg·L^−1^), *C* is the concentration of Cd^2+^ in the adsorbed solution, *V* is the volume of the Cd^2+^ solution (L), and *m* is the dosage of the composite aerogel beads (dry weight: g) [4].

#### 2.5.6. The Specific Surface Area Change of Cd^2+^ Adsorbed Composite Aerogel Beads

The Cd^2+^ adsorbed composite aerogel beads were substituted with alcohol (1 h, three times) and *tert*-butanol (1 h, three times), and then freeze-dried for 72 h. Afterwards, the composites were dried at 80 °C for 3 h. Then, the structural characteristics of the composites were determined by nitrogen gas adsorption at 77 K, using a BET surface area and pore size analyzer (Belsorp-mini II, BEL Japan Inc., Japan). Specific surface area (*S*_BET_), total pore volume (*Vt*), and pore diameter (*Dp*) of the composites were calculated by the nitrogen adsorption isotherm. Total pore volume was computed from the data at a relative pressure (*P*/*P_O_*) of 0.99. Average pore volume was calculated using the following formula:

*Dp* (nm) = 4*Vt* × 1000/*S*_BET_. (4)

#### 2.5.7. XRD Analysis on Cd^2+^ Absorbed Composite Aerogel Beads

The Cd^2+^ adsorbed composite aerogel beads (RC, RCCA-5, and RCNC-5) were freeze-dried for 48 h. The dried composite aerogel beads were analyzed using X-ray diffractometer (Ultima IV, Rigaku, Tokyo, Japan) with the Cu Kα line. The operation of XRD patterns worked at a 40 kV and 30 mA in the angular range from 5° to 90° with a step of 0.02°.

## 3. Results Discussion

### 3.1. Physical Analysis of Composite Aerogel Beads

#### 3.1.1. BET Analysis

**Figure 1:**
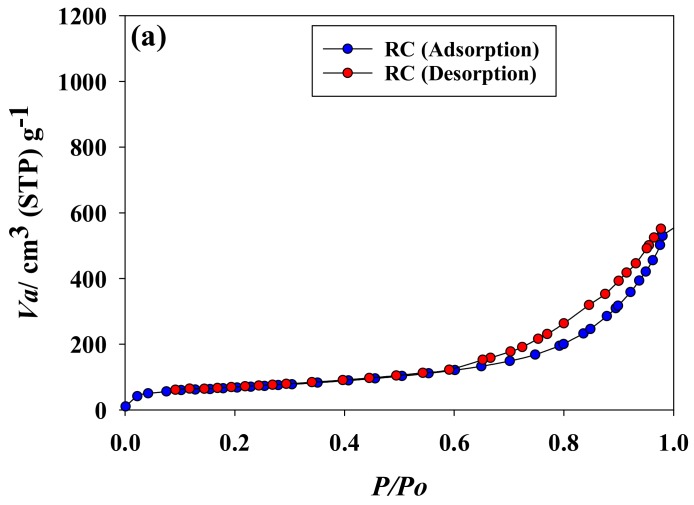

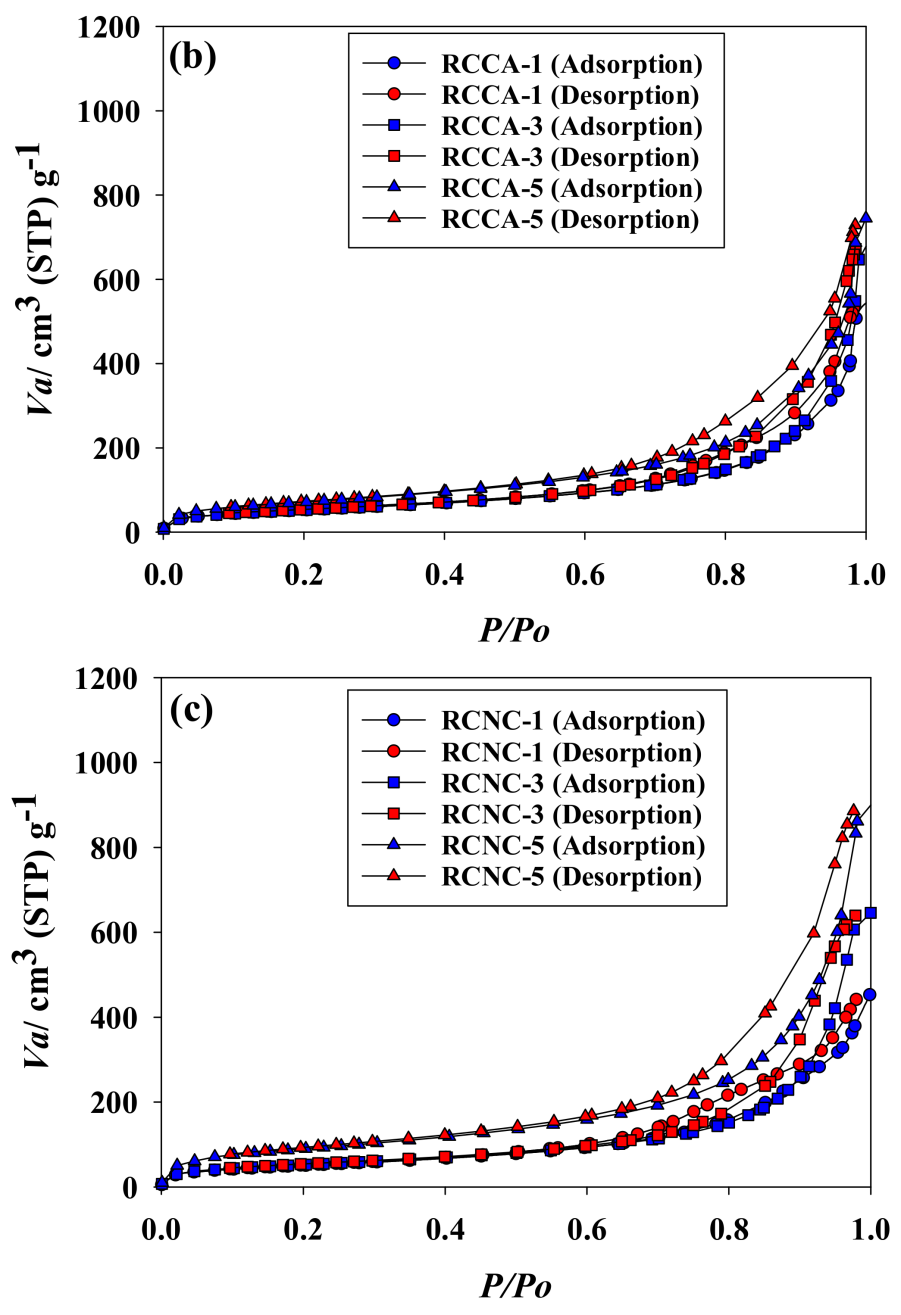
Nitrogen gas adsorption and desorption isotherms of (**a**) RC (regenerated cellulose) and the manufactured composites (**b**) RCCA-(1, 3, and 5), and (**c**) RCCA-(1, 3, and 5).

The nitrogen adsorption–desorption isotherms of RC, RCCA-(1, 3, and 5), and RCNC-(1, -3, and -5) showed increasing nitrogen adsorption as the relative pressure of nitrogen gas was increased (Figure 1). Moreover, the RCCA-(1, 3, and 5) and RCNC-(1, 3, and 5) microalgae-cellulose composites exhibited increasing nitrogen gas adsorption as the respective mixture ratio of *C*. *angulosa* and *N*. *commune* increased. Hence, the intricate porous attributes of the composite aerogel beads could be varied by adjusting the proportion of the green and blue-green algae powder used in the respective mixtures.

The same trend was observed from analysis of the specific surface area (Table 2). The specific surface areas of RCCA-(1, 3, and 5) were 189.89, 190.02, and 261.3 m^2^·g^−1^ and those of RCNC-(1, 3, and 5) were 183.15, 190.38, and 323.79 m^2^·g^−1^, respectively. Overall, BET showed the same tendency as the total pore volume because the number of pores increased as the ratio of microalgae increased [36]. The hydrogen bond is regenerated when the moisture inside the dissolved cellulose is removed by organic solvent substitution and drying. In the process, microalgae intercept into space, resulting in an increase of micro- and mesopores, which contributes to the increase of BET.

**Figure 2:**
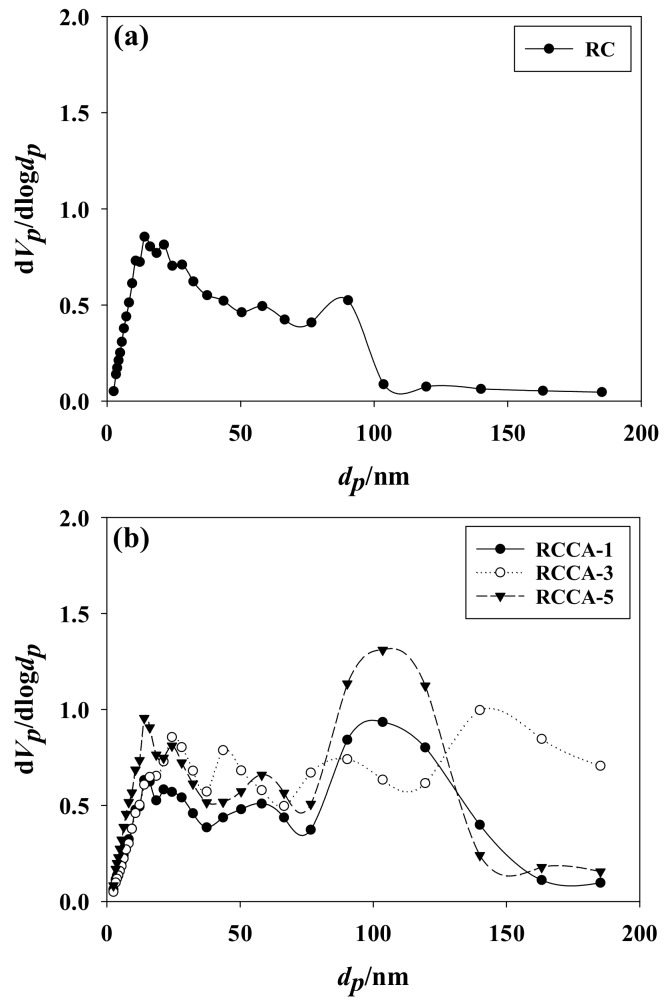

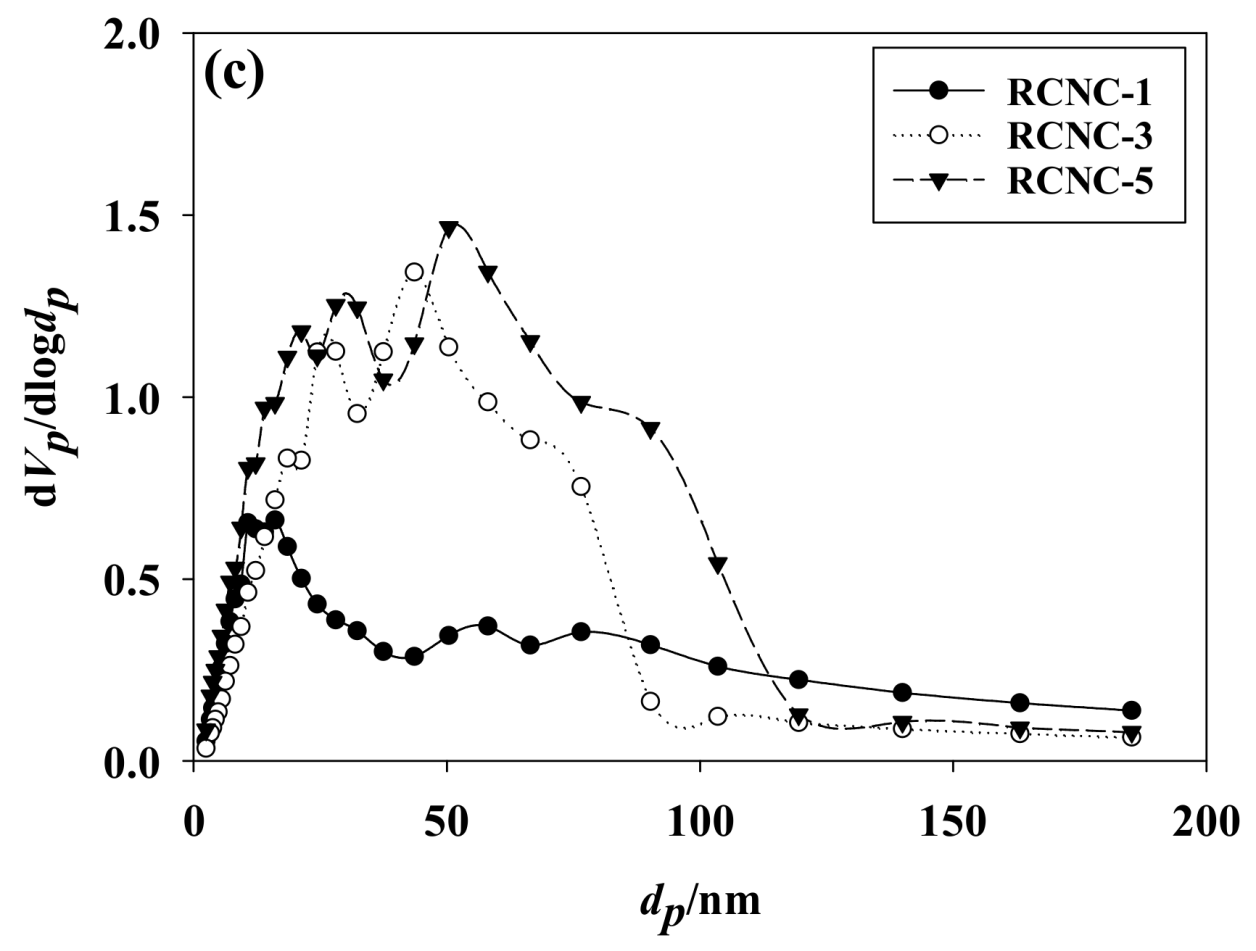
Pore size distribution of (**a**) RC and the manufactured composites, (**b**) RCCA (1, 3, and 5), and (**c**) RCNC (1, 3, ad 5).

The pores can be distinguished by the pore diameter (nm), with micropores <2, mesopores 2–50, and macropores <50. Figure 2 displays the pore distribution of the manufactured composites. All pore sizes (micro-, meso-, and macropores) were evident in RC, but micro- and mesopores were dominant. Gavilon and Budtova found that the bubbles occurring in the mixing process influence pores sizes. Micro- and mesopores seem to prevail in RC, judging from the absence of bubbles occurring at the mixing process [37]. The RCCA-(1, 3, and 5) composites were also prevalent with micro- and mesopores. However, macropores tended to prevail as the ratio of *C*. *angulosa* increased. The negative charge of microalgae and hydroxyl groups of cellulose block the aggregation of microalgae. The disposed microalgae remained as it was at regeneration and will be removed in the washing process, which leads to increase of macropores [38]. Conversely, micro- and mesopores predominated over the RCNC-(1, 3, and 5) composites, which means that they were comprised of more numerous small pores compared to RCCAs. The reason is that the cell walls of *C*. *angulosa* in RCCA-(1, 3, and 5) are rich in proteins and these proteins appear to dissolve under the strong alkaline conditions of LiOH that were used for the dissolution of cellulose [39,40]. However, *N*. *commune* in the RCNC-(1, 3, and 5) has a cell wall composed of peptidoglycan, which does not readily dissolve, effectively preventing the destruction of the cells from osmotic pressure under the alkaline conditions [41,42]. As a result, the increase in pore volume of the regenerated cellulose is prevented by *N*. *commune* cells.

#### 3.1.2. SEM Analysis

**Figure 3:**
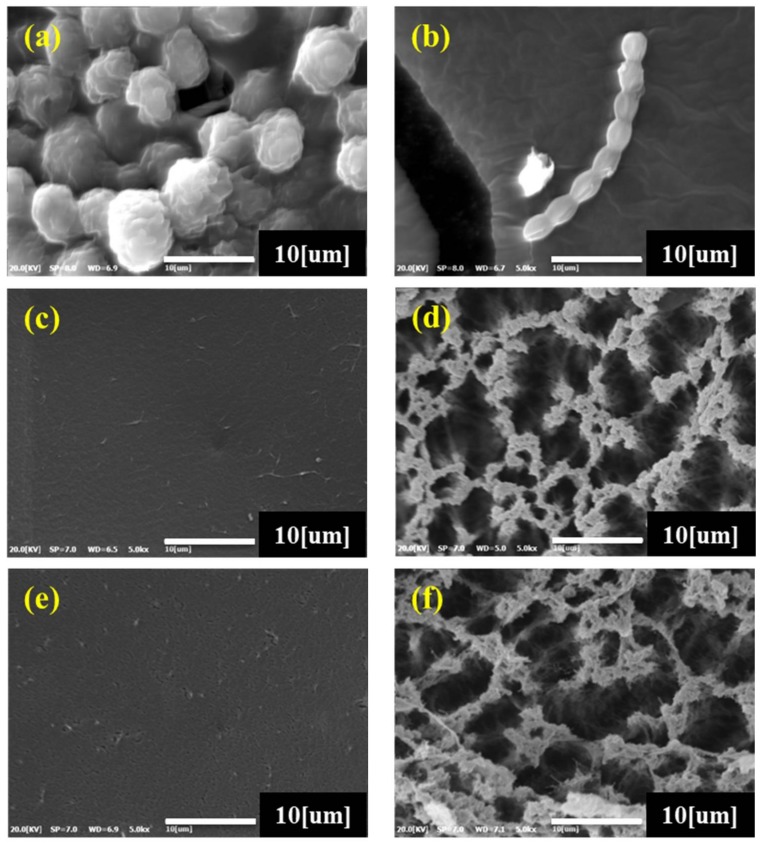

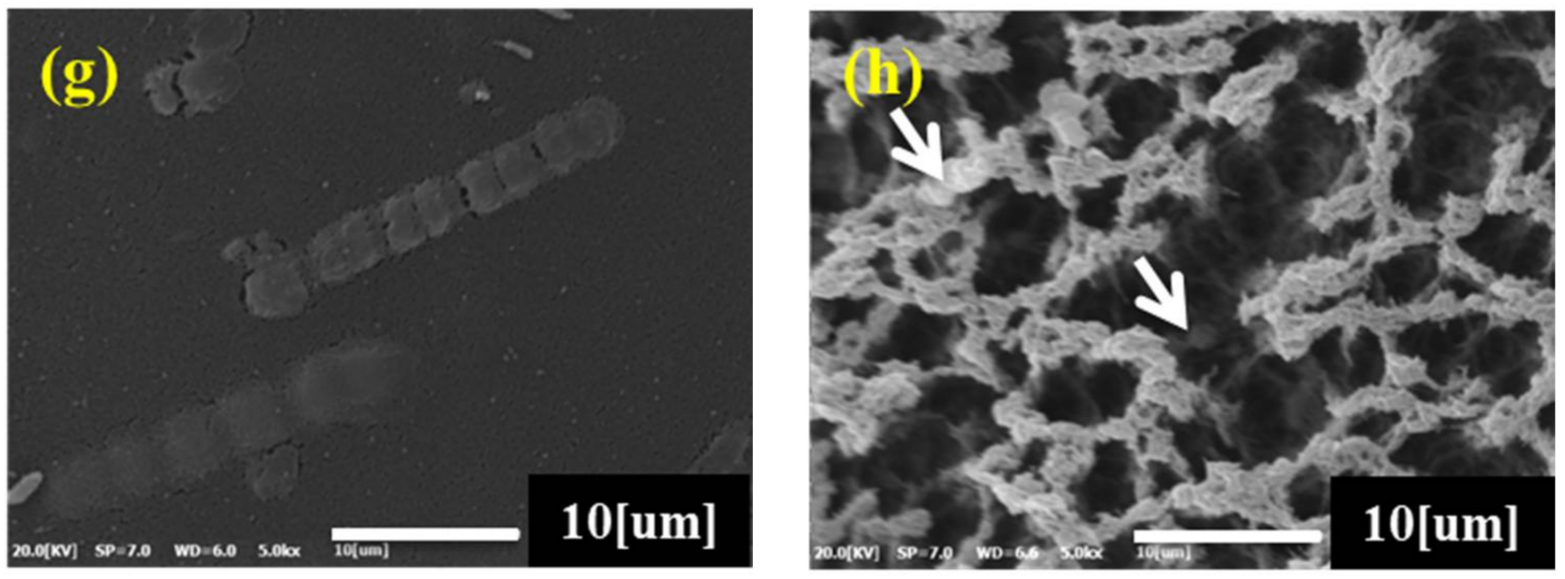
Scanning electron micrographs (×5000) of the raw materials, (**a**) *Chlamydomonas angulosa*, (**b**) *Nostoc commune*, (**c**) RC (surface), (**d**) RC (cross-section), and manufactured composites, (**e**) RCCA-5 (surface), (**f**) RCCA-5 (cross-section), (**g**) RCNC-5 (surface), and (**h**) RCNC-5 (cross-section).

The scanning electron micrographs of the surface and cross-section of *C*. *angulosa*, *N*. *commune*, RC, RCCA-5, and RCNC-5, respectively, are given in Figure 3. *C*. *angulosa* presented spherical cells (Figure 3a), and *N*. *commune* appeared as beads (Figure 3b). In the case of RC, a smooth surface was evident (Figure 3c), and the cross-section highlighted a porous network structure, formed by the dissolution and regeneration of cellulose (Figure 3d) [24,43]. This tendency was similarly confirmed in dissolution and regeneration research of cellulose using other alkali solutions. The porous network structure of RC was equally identified in the cross-sections of RCCA-5 and RCNC-5. However, *C*. *angulosa* was not observed either at the surface nor in the cross-section of RCCA-5, suggesting that the cells deteriorated in the alkaline environment, due to LiOH [39]. These results are consistent with other studies that showed that algae cell walls were destroyed when the cells were exposed to NaOH [41,42]. In contrast, *N*. *commune* cells were observed in the RCNC-5 cross-section, because, as mentioned above (Section 3.1.2), the peptidoglycan layer in *N*. *commune* is not degraded by the alkaline environment. Therefore, the cells are considered to exist between the regenerated cellulose fibers.

#### 3.1.3. Thermal Characteristics

**Figure 4:**
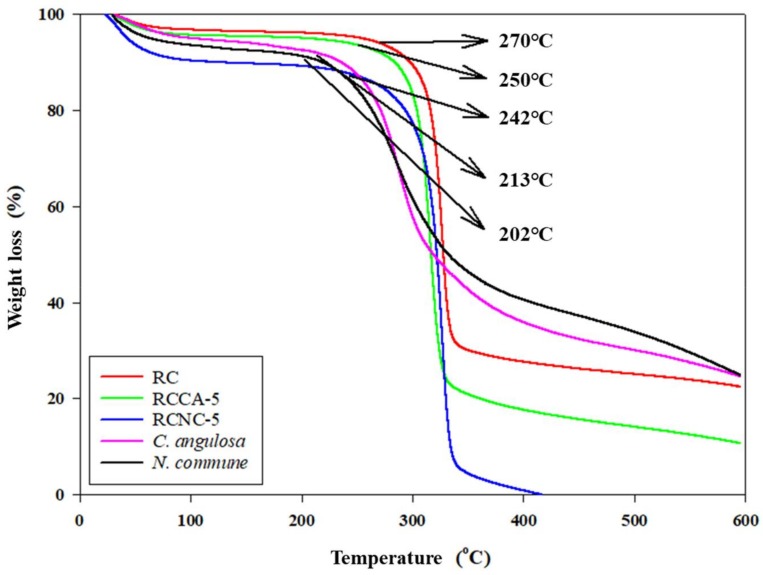
Thermogravimetric curves showing the decomposition of the raw materials (RC, *Chlamydomonas angulosa*, and *Nostoc commune*) and the manufactured composites (RCCA-5 and RCNC-5) at 10 °C/minute heating rate.

Figure 4 provides the TGA graph of RC, RCCA-5, RCNC-5, *C*. *angulosa*, and *N*. *commune*. The curves decreased gradually at 100 °C, due to evaporation of water from the manufactured composite aerogel beads. The weight loss of all of the samples was almost not achieved at 100–200 °C, and *N*. *commune* started to undergo pyrolysis at 202 °C. After that, rapid thermal decomposition of *C*. *angulosa* proceeded at 213 °C. The rapid pyrolysis of RC, RCCA-5, and RCNC-5 started at 270, 250, and 242 °C, respectively. Hence, the conditions facilitating rapid pyrolysis were changed by mixing thermally stable cellulose with the respective algae. The rapid pyrolysis of RC, RCCA-5, RCNC-5, *C*. *angulosa*, and *N*. *commune* was completed at a corresponding 340, 332, 326, 312, and 318 °C. The algae are mainly composed of proteins, carbohydrates, and lipids [44,45]. Protein is reported to undergo pyrolysis at about 200 °C [46], which corresponded with the results of this study. Cellulose possesses a high thermal stability, particularly the crystalline regions. Therefore, removing the inter- and intramolecular bonds of cellulose macromolecules requires a high pyrolysis temperature. In addition, the absence of hemicellulose and lignin seems to result in rapid pyrolysis over a narrow temperature range [47]. In this study, it was confirmed that the thermal stability of RCCA-5 and RCNC-5 was modified, due to the mixing of cellulose with algal cells.

### 3.2. Chemical Analysis of the Composite Aerogel Beads

#### 3.2.1. FTIR Analysis

**Figure 5:**
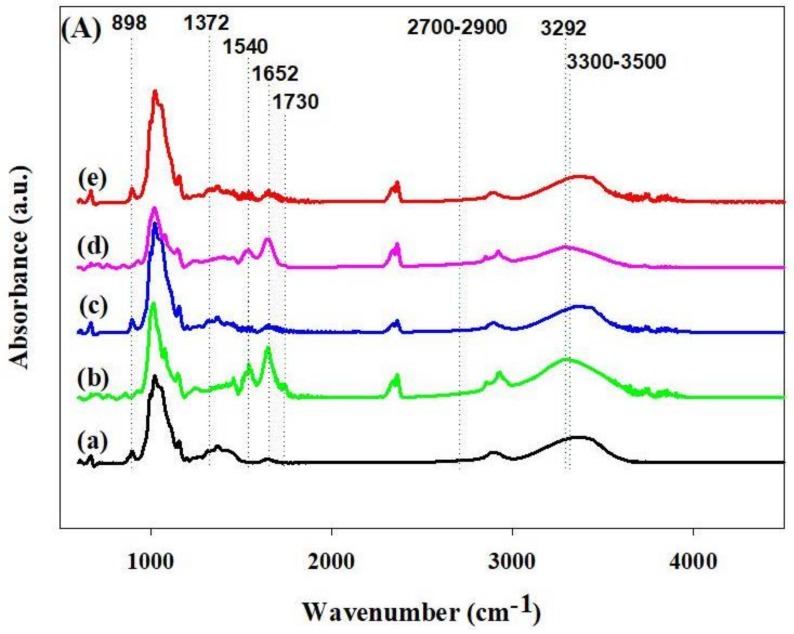

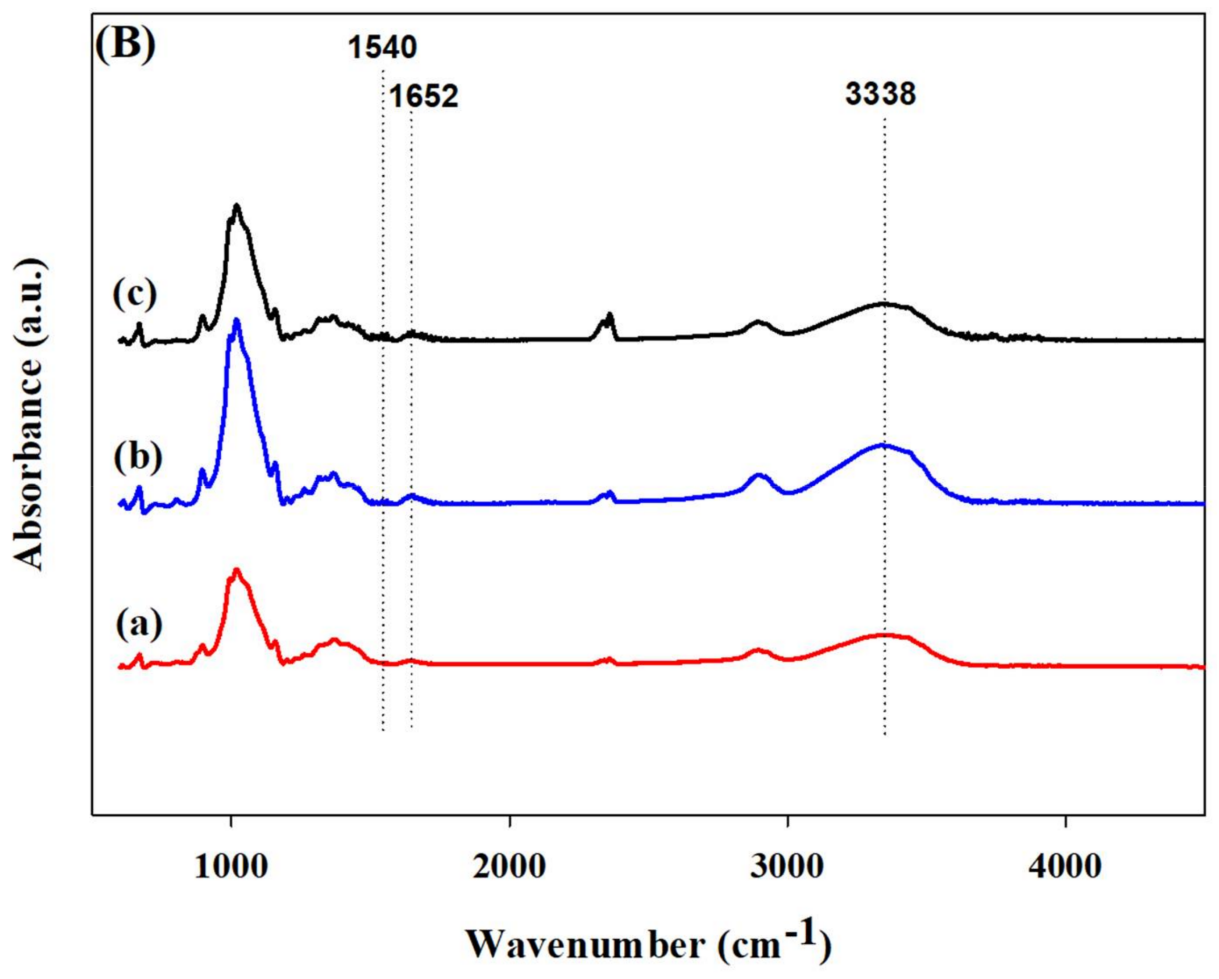
FTIR analysis of the composites and raw materials (**A**): (**a**) RC, (**b**) *Chlamydomonas angulosa*, (**c**) RCCA-5, (**d**) *Nostoc commune*, and (**e**) RCNC-5. Furthermore, cadmium-adsorbed of composites (**B**): (**a**) RC, (**b**) RCCA-5 and (**c**) RCNC-5.

Figure 5A represents the chemical structures of RC, RCCA-5, RCNC-5, *C*. *angulosa*, and *N*. *commune* using FTIR. In RC, glycosidic C–H deformation, ring vibration, and O–H bending were observed at 898 cm^−1^. Furthermore, O–H bending vibration (1372 cm^−1^), –CH_2_ (2700–2900 cm^−1^), and stretching vibration of OH group (3300–3500 cm^−1^) was noted [48]. *C*. *angulosa* and *N*. *commune* showed signals originating from N–H (amide II, 1540 cm^−1^) and C=O (amide I, 1652 cm^−1^) groups. In addition, the presence of peaks corresponding to COOH (lipids and proteins, 1730 cm^−1^) and N–H (amide-A, 3292 cm^−1^) confirmed the presence of protein [49,50]. RCCA-5 and RCNC-5 showed protein peaks at 1540, 1652, and 1730 cm^−1^ from *C*. *angulosa* and *N*. *commune*, as well as OH stretching at 3300–3500 cm^−1^ from cellulose. The hydrogen bond between OH group from cellulose and N–H from microalgae affected changing of OH stretching peak of the composite. In addition, chemical bonds seem not to happen because peaks related to amide group (I, II) and COOH remain as they are. [23] The changes in carboxylate and hydroxylate peaks after Cd^2+^ adsorption were associated with the binding of cadmium ions and functional groups. It was confirmed that the carboxyl and hydroxyl groups were the major contributors for uptake of heavy metal ions [51].

#### 3.2.2. FTIR Analysis of the Cd^2+^-Adsorbed Composites

In the FTIR spectra of RCCA-5, RCNC-5, and RC after the Cd^2+^ adsorption test (Figure 5B), there was shift or disappearance of some peaks. These peaks corresponded to the N–H (amide II, 1540 cm^−1^), C=O (amide I, 1652 cm^−1^), COOH (lipids and protein, 1730 cm^−1^), and OH (hydroxyl, 3338 cm^−1^) groups. The intensity of the peak derived from the hydroxyl groups was particularly decreased. Furthermore, this same peak was observed to change from a sharp to a smooth curve. It is estimated that Cd^2+^ was effectively adsorbed by the functional amide, carboxyl, and hydroxyl groups belonging to *C*. *angulosa*, *N*. *commune*, and cellulose [4,52].

#### 3.2.3. Analysis of the Cd^2+^ Removal Characteristics

**Figure 6:**
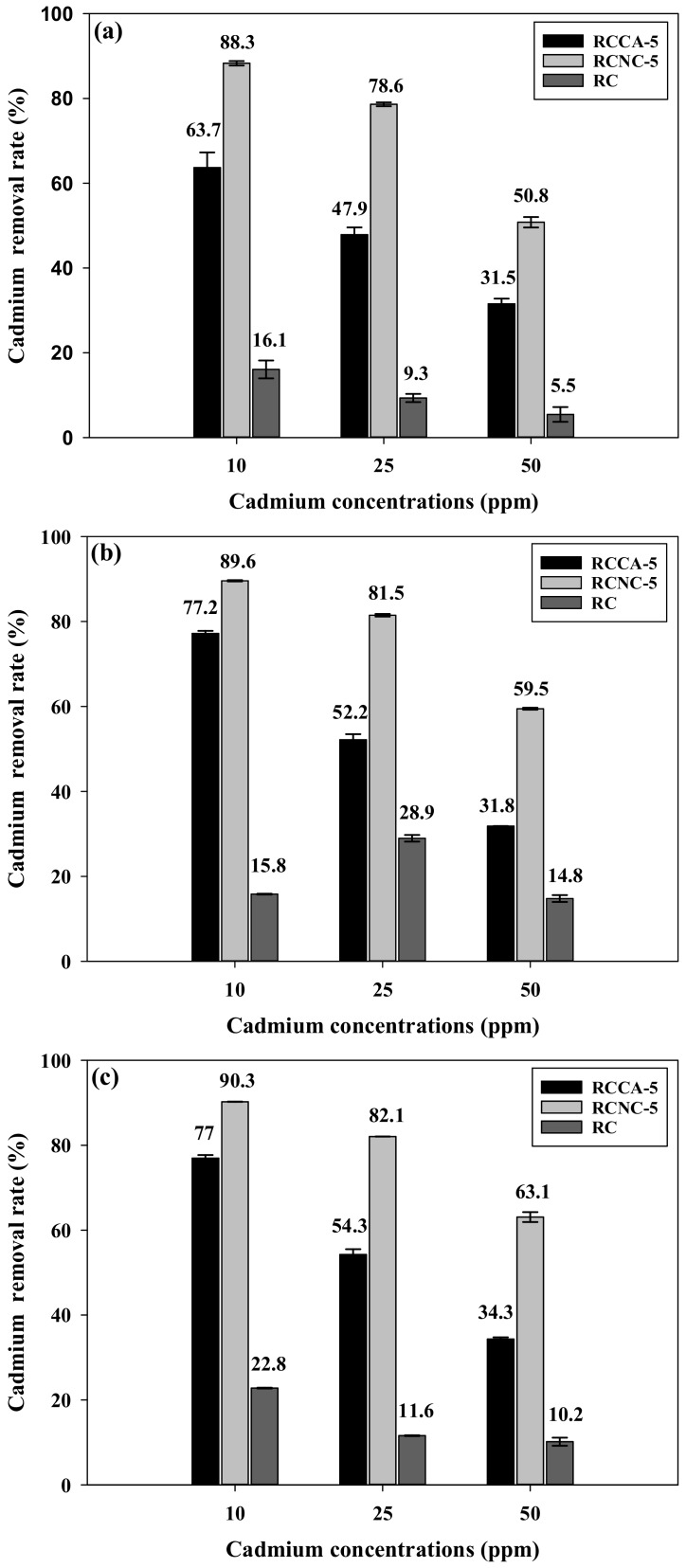
Cadmium removal rate by the manufactured composites (RCCA-5, RCNC-5) and RC, according to the heavy metal concentration at the reaction time of 120 min, at (**a**) pH 4, (**b**) pH 5, and (**c**) pH 6.

The Cd^2+^ removal rates, monitored by reacting Cd^2+^ solutions (10, 25, and 50 ppm) with RCCA-5, RCNC-5, and RC for 120 min, under pH 4, 5, and 6, are plotted in Figure 6. The RCCA-5 and RCNC-5 microalgae-cellulose composites showed a higher Cd^2+^ removal rate at all pH and Cd^2+^ concentrations than RC, and RCNC-5 presented the greatest removal rate. However, the removal rate decreased as the original Cd^2+^ concentration increased. The RCCA-5, RCNC-5, and RC showed a gradual increase in the Cd^2+^ adsorption quantity, when the pH was changed to 4, 5, and 6, respectively. Figure 6b 25 ppm showed a sudden increase of removal rate. It seems to result in percentage expression because the weight of adsorbed heavy metal increases as initial ppm increases in other samples. These results suggest that RCCA-5, RCNC-5, and RC are largely affected by the pH conditions of the solution because Cd^2+^ adsorption depends on the surface properties of the material and the chemical state of the heavy metal ions [53]. At pH 6, the Cd^2+^ removal rate by RCNC-5 was 90%, 82%, and 63% at a corresponding 10, 25, and 50 ppm Cd^2+^. With regards to adsorption, pH is an important regulatory parameter. The Cd^2+^ exists in the form of free ions under pH 8, and the metal adsorption is associated with the functional group of the cell wall and ionic substances. The adsorption at low pH consists preferentially in H^+^ rather than metal ions. In the process of adsorption, H^+^ decreases as the pH goes higher and anionic ligands increase, whereas the high pH can cause precipitation by combined metal with OH^−^. In addition, carboxyl groups represent deprotonated anion charge over pH 3–4, which is useful for metal adsorption [54,55].

**Figure 7:**
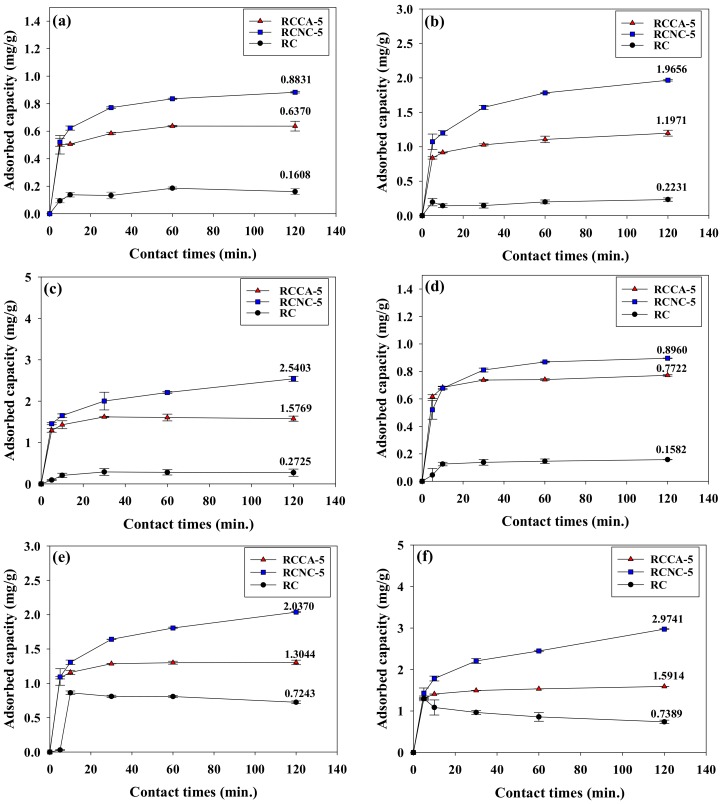

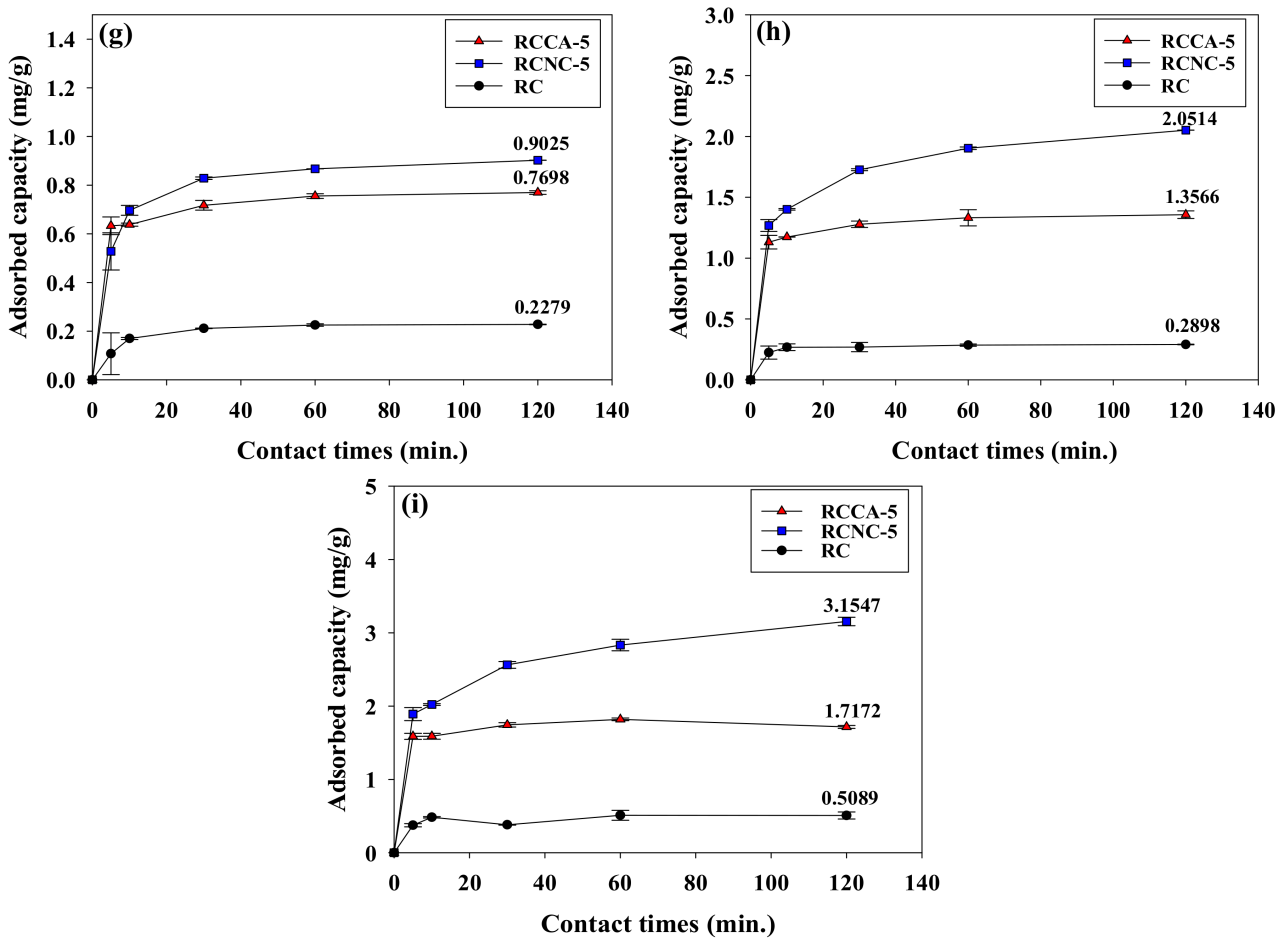
Cadmium adsorption by the manufactured composites (RCCA-5, RCNC-5) and RC, according to various pHs, times, and cadmium concentrations: (**a**) 10 ppm at pH 4, (**b**) 25 ppm at pH 4, (**c**) 50 ppm at pH 4, (**d**) 10 ppm at pH 5, (**e**) 25 ppm at pH 5, (**f**) 50 ppm at pH 5, (**g**) 10 ppm at pH 6, (**h**) 25 ppm at pH 6, and (**i**) 50 ppm at pH 6.

Figure 7 provides the Cd^2+^ adsorption (mg·g^−1^) per unit weight of RCCA-5, RCNC-5, and RC with time. At all pH and Cd^2+^ concentration conditions, the highest quantity of Cd^2+^ was adsorbed at the initial 5 min, which may be attributed to the empty interior spaces in the aerogel, which can easily absorb the solution. As the Cd^2+^ concentration increased, the adsorption capacity of RCCA-5, RCNC-5, and RC also increased. These results indicate that the higher the concentration of the heavy metal solution, the more the adsorption activity sites on the surface of the adsorbent can be effectively functionalized, thereby enhancing the chance to bond with the heavy metal ions [56]. The RCNC-5 at pH 6 exhibited the highest Cd^2+^ adsorption (mg·g^−1^), being 0.9025, 2.0514, and 3.1547 at 10, 25, and 50 ppm Cd^2+^, respectively. The results indicate that a high adsorption rate exists at a low initial Cd^2+^ concentration and a low adsorption rate occurs at a high initial Cd^2+^ concentration. Overall, the manufactured composites were found to remove Cd^2+^ effectively. Both microalgae-cellulose composites (RCCA-5 and RCNC-5) adsorbed more Cd^2+^ than RC. In particular, RNCN-5, the composite mixed with *N*. *commune*, presented more efficient removal of Cd^2+^ than RCCA-5, which was mixed with *C*. *angulosa*.

### 3.3. Properties of Adsorbed Cadmium on the Composite Aerogel Beads

#### 3.3.1. Variation of the Specific Surface Area

**Figure 8:**
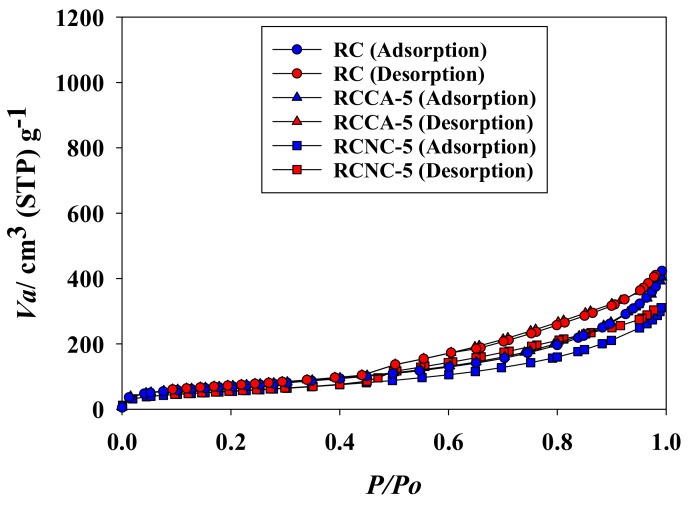
Nitrogen gas adsorption and desorption isotherms of composite aerogel beads after adsorption of Cd^2+^ ions.

**Figure 9:**
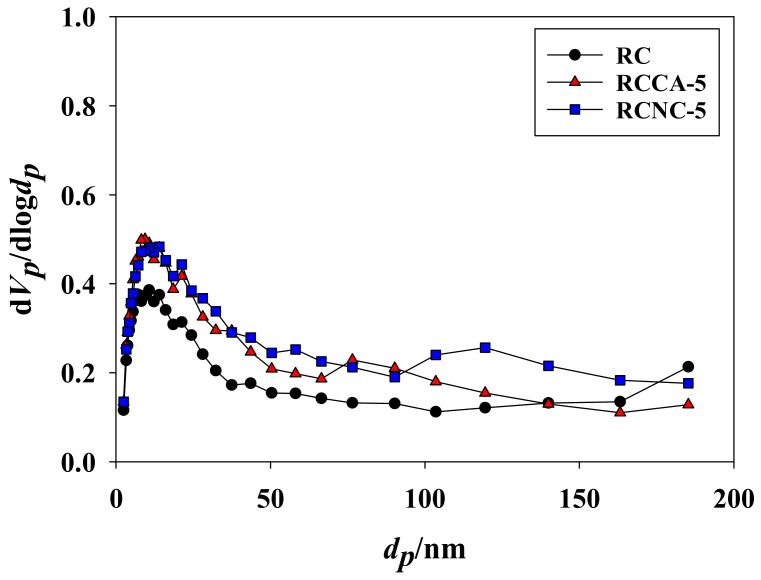
The pore size distribution of composite aerogel beads after adsorption of Cd^2+^ ions.

Table 3 shows the specific surface area of composite aerogel beads after Cd^2+^ adsorption at pH 6, and 50 ppm condition. The specific surface area, total pore volume, and mean pore diameter of composites (RC, RCCA-5, and RCNC-5) were decreased due to adsorption of Cd^2+^. In particular, the specific surface area of RCN-5 was significantly decreased to 254.40 m^2^·g^−1^ from 323.79 m^2^·g^−1^. Figure 8 is the nitrogen adsorption and desorption curves of composite aerogel beads after adsorption of Cd^2+^. The adsorption of nitrogen in Cd^2+^ adsorbed composites (RC, RCCA-5, and RCNC-5) show an increasing trend as pressure increases, although not significantly by adsorption of Cd^2+^. The most significant change was observed in pore sizes. RC, RCCA-5, and RCNC-5 have various pore sizes before Cd^2+^ adsorption; however, they were mostly meso- and micropores after Cd^2+^ adsorption (Figure 9). These results are similar to a result published by Xavier and Banda that a hysteresis loop appears in the adsorption isotherm along with the reduction of the specific surface area after adsorption of heavy metal. [57] Thus, the adsorption of Cd^2+^ was regarded as changing the pore characteristics of RC, RCCA-5, and RCNC-5.

#### 3.3.2. XRD Analysis of Adsorbed Cd^2+^ on the Composite Aerogel Beads

**Figure 10:**
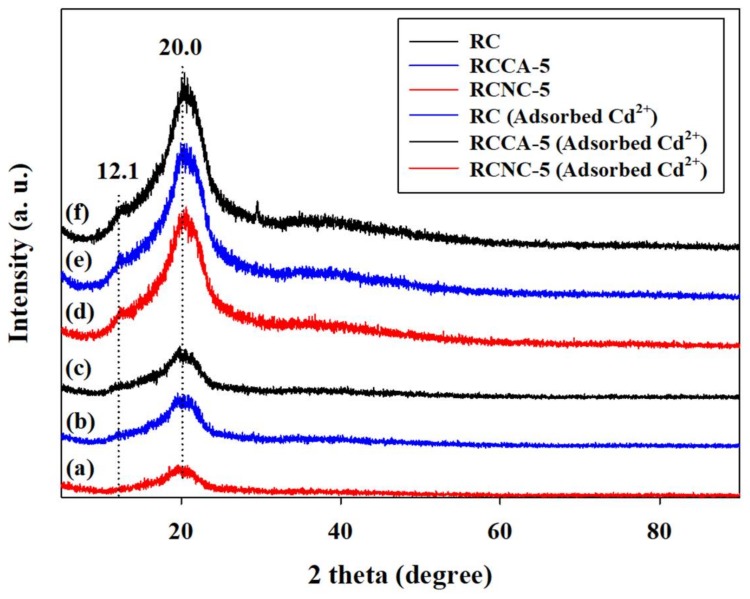
X-ray diffraction patterns of composites (RC, RCCA-5, and RCNC-5) before and after Cd^2+^ adsorption: (**a**) RCNC-5 (Adsorbed Cd^2+^), (**b**) RCCA-5 (Adsorbed Cd^2+^), (**c**) RC (Adsorbed Cd^2+^), (**d**) RCNC-5, (**e**) RCCA-5, and (**f**) RC.

Figure 10 is an XRD graph to analyze the crystallinity of RC, RCCA-5, and RCNC-5. All of the samples showed strong peaks at 12.1° (101), and 20.0° ($10\bar{1}$). When the cellulose is dissolved and regenerated by the LiOH/urea solvent, the molecular structure of cellulose is changed. The structure of cellulose was converted from cellulose (I) to cellulose (II) [58], whereas the XRD peaks of *C*. *angulosa* and *N*. *commune* were not observed due to the small amount of microalgae biomass. The adsorption of Cd^2+^ in RC, RCCA-5, and RCNC-5 did not shift the peaks, although the intensity of peaks decreased significantly. The reason is that the molecular structure of cellulose and microalgae of composites did not change by adsorption of Cd^2+^. These results are consistent with the study of Castaldi et al. and Sharma et al., in which the XRD peaks were not shifted while the intensity was decreased [59,60].

## 4. Conclusions

In this study, the physicochemical properties of manufactured composites prepared by mixing *C*. *angulosa* and *N*. *commune*, respectively, in dissolved cellulose, were analyzed. In order to investigate the removal of heavy metal ions, a Cd^2+^ adsorption test was carried out. In the BET analysis of the composites, the specific surface area of RCCA-5 and RCNC-5 increased as the mixing ratio of *C*. *angulosa* and *N*. *commune*, respectively, increased. SEM of RC, RCCA-5, and RCNC-5 revealed that the network structure of regenerated cellulose was present in all of the composites. In contrast to the *C*. *angulosa* cells in RCCA-5, however, *N*. *commune* cells were discovered in the internal network structure of RCNC-5. As a result of TGA, both *C*. *angulosa* and *N*. *commune* rapidly pyrolyzed at about 200 °C. Notably, cellulose-microalgae composites, RCCA-5 and RCNC-5, started rapid pyrolysis from 250 °C, which means that mixing the algal cells with cellulose changed the pyrolysis characteristics. FTIR analysis of the manufactured composites showed various functional groups representing *C*. *angulosa*, *N*. *commune*, and cellulose, with amide, carboxyl, and hydroxyl groups evident, due to the proteins, carbohydrates, and lipids present in the raw materials. Cd^2+^ adsorption characteristics of RCCA-5, RCNC-5, and RC at various pHs and Cd^2+^ concentrations were increased by the mixture ratio of the respective microalgae. RCNC-5 at pH 6 removed the heavy metal ions most effectively and at all Cd^2+^ levels studied (10, 25, and 50 ppm). The quantity of adsorbed Cd^2+^ (mg·g^−1^) per unit weight of RCCA-5, RCNC-5, and RC increased with increasing Cd^2+^ concentration. Moreover, RCCA-5 and RCNC-5 adsorbed more Cd^2+^ ions than RC, which did not adsorb any Cd^2+^ after 10 min, whereas RCCA-5 and RCNC-5 continuously adsorbed the ions for over 60 min. Finally, the signals corresponding to the amide, carboxyl, and hydroxyl groups shifted or disappeared in the FTIR spectra of RC, RCCA-5, and RCNC-5 after Cd^2+^ adsorption. For this reason, the adsorption of the Cd^2+^ ions was estimated to involve these various functional groups. In this study, manufactured composite aerogel beads using, respectively, *C*. *angulosa* (green alga) and *N*. *commune* (blue-green alga), efficiently removed heavy metal (Cd^2+^) ions from solution. The molecular structure of composites was confirmed to change in accordance with reducing the specific surface area, total pore volume, and mean pore sizes due to adsorption of Cd^2+^. XRD analysis, on the other hand, confirmed that adsorption of Cd^2+^ has absolutely no effect on the molecular structures of RC, RCCA-5, and RCNC-5.

## Figures and Tables

**Table 1:** Manufacture of composites according to the mixture ratio of cellulose and microalgae.

Sample Name	Mixture Ratio (100% (*w*/*w*))		
	Dissolved Cellulose (2%)		Microalgae
Regenerated cellulose (RC)	100	:	0
RC and *Chlamydomonas angulosa* (RCCA-1)	99.9	:	0.1
RC and *Chlamydomonas angulosa* (RCCA-3)	99.7	:	0.3
RC and *Chlamydomonas angulosa* (RCCA-5)	99.5	:	0.5
RC and *Nostoc commune* (RCNC-1)	99.9	:	0.1
RC and *Nostoc commune* (RCNC-3)	99.7	:	0.3
RC and *Nostoc commune* (RCNC-5)	99.5	:	0.5

**Table 2:** Specific surface, total pore volume, and mean pore diameter of manufactured composites.

Sample Name	BET Surface Area (m^2^·g^−1^)	Total Pore Volume ((*P*/*Po* = 0.990)/cm^3^·g^−1^)	Mean Pore Diameter (nm)
RC	235.71	0.48	14.22
RCCA-1	189.89	0.80	16.85
RCCA-3	190.01	1.00	21.08
RCCA-5	261.30	1.09	16.73
RCNC-1	183.15	0.65	14.26
RCNC-3	190.38	0.97	20.45
RCNC-5	323.79	1.36	16.81

**Table 3:** Specific surface, total pore volume, and mean pore diameter of composites after Cd^2+^ adsorption at pH 6, and 50 ppm condition.

Sample Name	BET Surface Area (m^2^·g^−1^)	Total Pore Volume ((*P*/*Po* = 0.990)/cm^3^·g^−1^)	Mean Pore Diameter (nm)
RC	201.67	0.47	9.40
RCCA-5	260.76	0.61	9.34
RCNC-5	254.40	0.64	10.02
